# Challenges of Alginate-Based Cast Films in Plastic-Free Food Packaging Applications: An Overview

**DOI:** 10.3390/polym17223061

**Published:** 2025-11-19

**Authors:** Sophie Schenk, Matthias Bucher, Michael Herrenbauer, Daniela Schmid, Markus Schmid

**Affiliations:** 1 Sustainable Packaging Institute SPI, Faculty of Life Sciences, Albstadt-Sigmaringen University, Anton-Günther-Str. 51, 72488 Sigmaringen, Germany; bucher@hs-albsig.de (M.B.);; 2 Technical Product Management—Technical Deisgn, Faculty of Printing and Media, Stuttgart Media University, Nobelstraße 10, 70569 Stuttgart, Germany; herrenbauer@hdm-stuttgart.de

**Keywords:** sodium alginate, food packaging, biopolymer films, crosslinking, plasticizers, barrier properties, processability

## Abstract

This review investigates the potential of sodium alginate, a biobased polysaccharide from brown algae, for food packaging applications. It analyzes the main challenges of cast films, including water vapor permeability, mechanical performance, and processability, and evaluates strategies to enhance these properties without chemical modification. Chemical modification is excluded because it would classify alginate as a plastic under EU regulations (PPWR, SUPD), conflicting with plastic-free packaging. The review synthesizes literature from 2004 to 2025 on pure sodium alginate films that are plasticized and ionically crosslinked without additional modifiers or nanofillers. While alginate provides excellent oxygen and fat barriers, its high water vapor permeability and brittleness limit broader use. Ionic crosslinking improves strength and water resistance, yet non-uniform networks remain a key challenge. Film performance is also influenced by drying temperature, mixing speed, molecular weight, and protein incorporation. This review differs from previous studies by highlighting the coupled effects of plasticization, ionic crosslinking, and processing limitations that together determine alginate’s industrial feasibility. Research gaps concern long-term stability and behavior under industrial packaging conditions. Given environmental and regulatory pressures to replace fossil-based plastics, sodium alginate shows strong potential as a scalable, renewable material for sustainable food packaging.

## 1. Introduction

Plastic packaging remains the most common material for food preservation [1]. Their barrier against gases and moisture, as well as their mechanical stability, protect the food from microbial and physicochemical spoilage. Packaging therefore makes a significant contribution to reducing food waste [2,3,4]. The growing demand for plastics goes along with the increasing use of finite resources. Not only the extraction of raw materials, but also the disposal of plastic waste after use poses a challenge to people and the environment [5,6]. In response, the European Union’s Bioeconomy Strategy promotes sustainable growth such as the development of renewable alternatives to fossil-based materials. One promising alternative to conventional plastics is the use of bio-based materials, with algae-derived polymers gaining increasing attention due to their sustainability and availability [7]. Among these, the polysaccharide that has recently been of interest for its potential in various applications is sodium alginate [8,9,10,11]. Sodium alginate is extracted from different types of brown algae, which are abundantly available in marine ecosystems [12].

Currently, alginates are applied in various industries, notably in the pharmaceutical industry such as tissue engineering, drug delivery, as well as in the food industry as a thickening and gelling agent [13,14]. In the food industry, alginates are also used in film applications, particularly by sausage manufacturers for the production of sausage casings [15]. Alginate is also used in sports nutrition to encapsulate carbohydrate liquids [16]. Beyond these established applications, recent research is also focusing on the production of alginate films for packaging applications due to its ability to form biodegradable and biocompatible films. Sodium alginate can form a continuous, stable matrix that produces tasteless, odorless, and transparent films, making it particularly suitable for film production. The films also have a much higher barrier effect against oxygen and carbon dioxide than commercially available films such as polylactic acid and show a high fat barrier [9,10,12,17,18,19,20,21,22,23]. But the hydrophilic nature of alginate results in high water vapor permeability and water solubility, limiting its functionality in moisture-sensitive applications [24,25,26]. Furthermore, unlike conventional plastic films, such as polyethylene, alginate films are produced by random polymerization, which leads to structural inhomogeneity and lower mechanical properties [24,25,26]. Although cross-linking with multivalent cations such as Ca^2+^ can enhance water resistance and mechanical integrity, the process also presents challenges, including non-uniform crosslinking, film shrinkage induced by the cross-linking process and a lack of compatibility with conventional thermal processing methods such as sealing or thermoforming [13,26,27,28,29].

In addition to providing barrier functionality, food packaging must fulfill further industrial requirements. These include sealability, moldability, printability, and resistance to mechanical stress during processing, transport, and storage [30,31,32]. Therefore, it is imperative that packaging films exhibit both suitable material properties and reliable performance in existing industrial processing and filling processes.

This review provides a narrative overview of current research (2004–2025) on sodium alginate films for food packaging. It focuses on the challenges of alginate films, in particular, water vapor permeability (WVP), mechanical stability and processing limitations, and evaluates current approaches to improve these properties through plasticization, cross-linking, and process adaptation. The review is structured according to the physical and barrier properties of alginate films. For each property, the influence of plasticizers and cross-linking agents is analyzed. Regarding crosslinking agents, the focus is on calcium chloride (CaCl_2_) because calcium is physiologically safe and, unlike iron, does not cause discoloration of the films [33,34,35]. This review only discusses percentage changes in film properties, while exact values are summarized in overview tables at the end of each chapter. Furthermore, studies with chemically modified alginate were deliberately excluded, as chemical modification would categorize alginate as plastic under the EU regulation (PPWR, SUPD), which would contradict the aim of developing plastic-free packaging solutions and ensuring the applicability of the results for the EU market. The review also discusses industrial requirements and the potential use of alginate films in practical applications. The objective of this study is to identify the key limitations of alginate-based films with the aim of facilitating the translation of research findings into industrial practice and establishing sodium alginate as a viable large-scale alternative to conventional plastic films. Contrary to previous reviews, this study emphasizes the interdependence of plasticization, ionic crosslinking, and processing constraints. These constraints are currently hindering the industrial viability of alginate-based films.

## 2. Alginate Properties and Applications

Alginate is a natural polysaccharide derived from brown seaweed. The chemical composition and structural characteristics of alginate exhibit variability depending on the algal source [36]. Chemically, alginate can be characterized as a salt of alginic acid. The alginic acid forms a block copolymer of β-D-mannuronic acid (M) and α-L-guluronic acid (G) residues. The blocks are covalently linked together and can be arranged as homopolymeric G-blocks or M-blocks or alternating heteropolymeric GM-blocks [37]. The structural design of the two units is the reason for the M-blocks’ flexible properties and the G-blocks’ rigid properties [38]. Therefore, the ratio of the M and G units and their arrangement within the polymer chains are decisive for the characteristic properties of alginate [39]. In particular, the geometry of the G-blocks is central to the gelation of alginates, as it creates electronegative cavities for the binding of di- or trivalent ions [40]. The stabilization of these egg-box dimers takes place via hydrogen bonds [41]. The process of ionic bonding is known as cross-linking [42]. As a result of these molecular interactions, the mobility of the alginate chains and thus the flexibility of the structure are restricted. This is why pure alginate films tend to be brittle [43].

Alginates have a high Young’s Modulus; however, their Tensile Strength and Elongation at Break are low [44]. Therefore, plasticizing additives are required during the production of alginate films. Depending on the desired properties of the films, hydrophilic plasticizers like sorbitol and glycerol and hydrophobic plasticizers like oils or fatty acids can be used [45].

Currently, investigations are strongly focused on food preservation, as the alginate microstructure gives alginates a barrier function, particularly against oxygen and fat, thus preserving food through stabilizing [8]. Alginates have a high oxygen (0.48 × 10^−6^ mL·mm/(m^2^·day·Pa) [46]) and fat barrier, but due to the hydrophilic nature of the material, the WVP is high (2592–2.68 × 10^−5^ g·m/(m^2^·day·Pa) [19]) and alginate films in contact with water swell [47,48,49]. As a result, the films act as selective barriers to water, gas, and other external compounds [50]. A well-balanced barrier function is therefore particularly necessary for the use of alginate films in food packaging to prevent spoilage [28].

Alginates are heat-stable when cross-linked, so they have no thermoplastic properties and cannot be sealed without further additives [13,27]. Moreover, several studies emphasize that pure alginate films present considerable challenges when sealing due to their lower strength and elasticity compared to conventional plastic films [15,27,45]. In addition, the thermal stability of the material also limits its application in thermo-forming. The mechanical performance and thermal stability of alginates are inadequate for such processes [51]. Conversely, the dimensional stability of the material is constrained, signifying that pure alginate might not possess sufficient stability for utilization in processes such as thermoforming [52]. Differential scanning calorimetry (DSC) analyses show that sodium alginate exhibits a broad endothermic peak at around 100 °C, corresponding to the loss of bound water, and a sharp exothermic peak between 240 and 260 °C, indicating thermal decomposition without prior melting. This behavior confirms the absence of a thermoplastic transition and makes pure alginate unsuitable for conventional thermoforming processes that require controlled melting below the decomposition temperature [53,54]. Another challenge for industrial applications is the sealing of alginate films. Since sodium alginate does not exhibit thermoplasticity, conventional heat sealing cannot be used without the addition of additional components. In addition, the sealing areas are often the weakest points in the packaging structure, where failure occurs due to the inherently low strength and elasticity of alginate compared to synthetic polymers. To overcome these limitations, researchers have investigated blends containing other biopolymers, as well as protein-based adhesives such as carboxymethylcellulose and gelatin [27]. Furthermore, ionic crosslinking with calcium can enhance the water resistance and tensile strength of alginate films. However, this process concomitantly reduces flexibility, a factor that can further complicate processing in sealing applications [28]. However, as this study focuses on pure alginate films rather than blends, these modification strategies will not be considered further.

In the ensuing sections, an analysis is conducted of the challenges and advantages associated with the utilization of pure alginate films in the packaging sector.

## 3. Physical Properties

In the ensuing sections, it is imperative to acknowledge the interdependence of the mechanical properties and barrier properties of alginate films, as their optimization invariably exerts a reciprocal influence on each other. This effect is particularly evident in the case of plasticizers. As already mentioned, plasticizers are typically added to alginate films to compensate the brittleness of the material. The use of plasticizers can increase the elasticity of the material by reducing the intermolecular bonding. However, this results in a loosening of the network, which makes the film more permeable to gases. For instance, Jost et al. (2014) observed that adding 40 wt% glycerol to sodium alginate films increased the elongation at break from about 10% to 35%, while simultaneously raising the oxygen permeability from approximately 0.35 to 1.8 cm^3^(STP)·100 µm/(m^2^·day·bar) [47]. Therefore, attempts are made to optimize the plasticizer content for optimum flexibility of the film [10,45,47].

Furthermore, cross-linking agents such as calcium ions can increase both mechanical strength and water resistance of alginate films. This creates the previously described egg-box structure, which in this case simultaneously improves the barrier properties. Jost and Reinelt (2018) demonstrated that CaHPO_4_-crosslinked films exhibited a significant barrier improvement, with WVP decreasing from 6.62 × 10^−7^ g·m/(m^2^·day·Pa) to 3.79 × 10^3^ g·m/(m^2^·day·Pa) and oxygen permeability (OP) decreasing from 1.86 × 10^−13^ g·m/(m^2^·day·Pa) to 1.06 × 10^−13^ g·m/(m^2^·day·Pa) [55]. However, the cross-linking of the films can concurrently result in the formation of cracks on the surface, which reduces the gas barrier of the films [29,42,56,57,58,59,60].

This shows that the subsequent crosslinking and plasticizing of alginate films in particular have a decisive influence on the final film properties, especially if targeted additivation, for example, with nanoparticles, is to be avoided. The presence of individual additives has been shown to induce alterations in material properties, thereby complicating the direct evaluation of the fundamental challenges associated with alginate films [44]. In addition, pure biopolymers are often of interest in the packaging industry for economic and regulatory reasons, as fewer additives allow for easier authorization and more practical industrial processing [61,62]. Furthermore, a detailed examination of specific additives for active and intelligent packaging applications would focus on the interactions between the additives, the packaged food, and the alginate. Such an analysis would complicate the comparison of results and exceed the scope of this review. Therefore, the focus will be placed on alginate and its interactions with copolymers, plasticizers, and crosslinkers.

### 3.1. Mechanical Properties

#### 3.1.1. Influence of Plasticizers and Biopolymer Blends

The mechanical properties of alginate films can be affected by the addition of plasticizers, which alter the flexibility and strength of the material (summarized in Table 1). Plasticizers disrupt the polymer network, increase molecular mobility and reduce intermolecular forces. Glycerol is one of the most commonly used plasticizers for alginate films due to its strong hydrogen bonds with the alginate chains. It contains three hydroxyl groups, which form strong hydrogen bonds with alginate chains, thereby increasing flexibility and reducing brittleness [63]. Additionally, glycerol is easy to mix with alginate solutions and does not cause phase separation, making it an effective plasticizing agent [34]. However, glycerol tends to migrate and evaporate over time, leading to a gradual loss of flexibility and increased brittleness of the film [45,64]. Glycerol is generally considered a non-volatile plasticizer due to its high boiling point. However, studies have shown that even at room temperature (25 °C), approximately 10% of glycerol can be lost during film drying (at 25 °C: approx. 0.452 g/g; at 90 °C: approx. 0.369 g/g) or during storage (no quantitative data available) [47,65,66]. This limits the long-term stability of alginate films plasticized with glycerol and poses a challenge for maintaining consistent mechanical properties in packaging applications [10,67].

In contrast to glycerol, hydrophobic plasticizers such as tributyl citrate and oils can lower the WVP and the tendency of the polymer to absorb water from the environment [45]. However, resulting films plasticized with tributyl citrate showed an increase in tensile strength and, unlike hydrophilic plasticizers such as glycerol, a slight reduction in elongation at break [10]. Similar effects have been observed with other water-insoluble plasticizers, such as oregano essential oil (OEO) [68]. Vegetable oils primarily act by blocking free voids in the polymer network, thereby reducing the water vapor transmission rate (WVTR). Oils interfere with the cohesion between polymer chains, thereby weakening the film structure [45]. In alginate-based films, the addition of OEO also led to a marked reduction in tensile strength, up to 56%, due to weakened cohesive forces between polymer chains. However, in contrast to tributyl citrate, the presence of dispersed oil droplets enhanced the flexibility of the films, resulting in a noticeable increase in elongation at break [68]. These findings indicate that while both types of hydrophobic plasticizers can effectively reduce water vapor permeability and tensile strength, their effects on film flexibility vary considerably depending on their molecular structure, interaction with the polymer, and physical state within the matrix.

Furthermore, achieving an optimum plasticizer concentration is important, as both too high and too low quantities can have a negative impact on the mechanical properties of alginate films [69]. If too little plasticizer is added, an anti-plasticization effect can occur. With the addition of plasticizer, the free volume in the amorphous areas of the polymer increases, which leads to an increase in crystallinity. As a result, the material becomes stiffer and the tensile stress and elongation at break are higher [70]. This effect was observed with different plasticizers such as polyglycerol, glycerol, sorbitol, xylitol, and fructose, if less than 7% (*w*/*w*, dry basis) has been added [45,67,71].

However, at higher plasticizer concentrations (e.g., above 14–25%), the effect is reversed: the tensile strength decreases and the typical plasticizer effect dominates, increasing the flexibility of the material [45,69]. This emphasizes the need for precise control of the plasticizer content in order to achieve a balance between the strength and flexibility of alginate-based films.

In addition to conventional plasticizers, mixing alginate with other biopolymers can also contribute to greater flexibility. Polymers such as lignin, pectin, starch or chitosan introduce additional molecular interactions that change the mechanical behavior of alginate films [45]. In this context, Gohil et al. discovered that blends of NaAlg and pectin with up to 40% pectin exhibited enhanced mechanical properties compared to pure alginate. Treatment with CaCl_2_ created physical crosslinks in the polymer network, altering the film structure. Higher pectin content made the films more flexible, and this positive effect was maintained even after CaCl_2_ treatment in blends containing up to 20% pectin [72].

Furthermore, it has been shown that alginate copolymer films containing 0.25% potato starch have a significantly higher elongation at break. This is attributed to reduced interactions between the alginate chains, which enable increased molecular movement and greater flexibility. The moderate increase in elongation at break of around 15% is particularly important, as alginate films remain brittle even when plasticizers and crosslinkers are used due to the strong intermolecular interactions and the limited mobility of the polymer chains [15].

Several studies have also investigated mixtures of sodium alginate and chitosan that form polyelectrolyte complexes. Meng et al. reported that these mixtures exhibit significantly different mechanical properties compared to pure alginate films. At a 50:50 alginate-to-chitosan ratio, the tensile strength increased by about 18% (44.28 MPa to 37.57 MPa), the elongation at break decreased by about 95% (4.36% to 90.55%), and the elastic modulus decreased by about 64% (1900 MPa to 4774 MPa). These changes were attributed to the formation of ionic and hydrogen bonds between the polymers, which altered the crystallinity and molecular orientation within the film [73]. Wibowo et al. confirmed the general trend that higher chitosan content in Ca^2+^-crosslinked alginate films increases tensile strength, decreases elongation at break, and lowers water vapor permeability [74].

Temperature conditions also influence the effectiveness of plasticizers in alginate films. Castro-Yobal et al. found that films containing glycerol and CaCl_2_ crosslinking remained flexible and elastic even at low temperatures (approx. −50 °C to 4 °C). However, too high plasticizer concentrations can negatively impact stability. To maintain mechanical integrity, it was observed that glycerol content should remain below 10% (*w*/*v*) [67,75].

**Table 1:** Effect of plasticizers on the tensile strength, Young’s modulus and elongation at break of sodium alginate films.

Plasticizer/Blend	Effect on Tensile Strength	Effect on Young’s Modulus	Effect on Elongation at Break	Reference
Glycerol	Initially ↓ with time ↑ brittleness due to loss of glycerol	Initially ↓ with time ↑ brittleness due to loss of glycerol		[45,47,64,65,66]
Oils	↓ by 56% (1.5% OE)	↑ by 68.2% (1.5% OE)		[68]
Tributyl citrate	↑ by 42.4% compared to Glycerol	↓ by 45.5% compared to Glycerol		[10]
Pectin	↓ by 5% (from 61.3 MPa with 0% Pectin to ~58 MPa with 20% Pectin)	↓ by 8% (from 3.68 GPa with 0% Pectin to ~3.4 GPa with 20% Pectin)	↑ by 67% (from 6% with 0% Pectin to ~10% with 20% Pectin)	[72]
Chitosan	↑ by ~18% (from 37.57 MPa with 0% Chitosan to 44.28 MPa with 50% Chitosan)	↓ by ~60% (from 4 774 MPa with 0% Chitosan to 1 900 MPa with 50% chitosan)	↓ by ~95% (from 90.55% with 0% Chitosan to 4.36% with 50% Chitosan)	[73]
Potato starch			↑ by 15% (0.25% starch) compared to pure alginate films	[15]

↑: increases; ↓: decreases.

#### 3.1.2. Influence of Crosslinking

Crosslinking affects the tensile strength, elongation at break and overall stability of the film (summarized in Table 2). Liling et al. conducted a study to examine the impact of diverse ionic crosslinking parameters on the mechanical properties of alginate films. Their research examined the effects of both divalent (Ca^2+^, Zn^2+^, Mn^2+^) and trivalent (Al^3+^) cations, as well as different concentrations (1–5% *w*/*v*) and crosslinking durations (1–6 min) of calcium chloride (CaCl_2_) as the crosslinking agent [29]. As the Ca^2+^-crosslinked films demonstrated the highest tensile strength (TS) of approximately 135 MPa and the greatest elongation at break, the crosslinking behavior of calcium will be the primary focus. The concentration of the crosslinking solution has a significant influence on the mechanical properties of alginate films. According to Liling et al., the optimal calcium concentration for maximum tensile strength and elongation was 2%. As the concentration increased, a decline in mechanical performance was observed. One possible explanation for this is the acceleration of the crosslinking reaction with increasing Ca^2+^ concentration. In the presence of excess calcium, crosslinking occurs rapidly at the film surface and forms a barrier that hinders the further diffusion of Ca^2+^ ions into the film interior. As a result, deeper regions of the film remain insufficiently crosslinked, leading to weaker overall mechanical properties [28,29,76].

The duration of the cross-linking process is also important for mechanical strength. Studies show that tensile strength increases with longer cross-linking times, while the elastic modulus increases up to 2 minutes and then decreases again with longer cross-linking times [29]. The reason for the first increase in the Elongation at Break value is attributed to the theory of free volume in calcium cross-linking. Calcium ions are characterized by their selective cross-linking behavior. Cross-linking only takes place with the G-blocks, which according to Straccia et al. and Russo et al. results in a coarse-meshed network [77,78]. Due to the selective cross-linking behavior of the calcium ions, the network cannot be packed more tightly. After about 2 minutes, the cross-linking reaction reaches saturation. Beyond this point, prolonged cross-linking leads to two effects: the glycerol present in the cross-linking bath begins to dissolve due to interactions with the coarse-meshed network, and the network structure destabilizes, reducing film elasticity and mechanical integrity [29].

The coarse-mesh structure of calcium-crosslinked alginate films also influences their thickness and shrinkage behavior. Due to the film’s high hydrophilicity, some of the alginate dissolves or is leached out during the crosslinking process, leading to increased shrinkage [29,79].

Furthermore, Sharmin et al. showed that citric acid initially acts as a crosslinker, enhancing the structural integrity and reducing the water vapor transmission rate. At higher concentrations (1% and 2% *w*/*v*), the plasticizing effect dominates instead, decreasing tensile strength by 58% and 66% while increasing elongation at break from 4% to 15% and 23% [48].

Moreover, it is also important to note that different crosslinking agents affect shrinkage rates differently. For instance, Al^3+^, as a trivalent ion, forms a denser crosslinking network due to its capacity to form more extensive coordination bonds within the polymer network. This results in reduced shrinkage because less alginate is lost during the process. In contrast, Calcium ions (Ca^2+^) result in a more porous network, thereby promoting greater shrinkage due to increased water interaction [29]. Although crosslinking enhances the barrier properties of alginate films, high water sensitivity remains a salient characteristic. Despite various crosslinking approaches, achieving complete water resistance remains a challenge as it often compromises other essential material properties [55].

For food packaging applications, effective cross-linking of the alginate films is required and ensuring uniform distribution of cross-linking agents is crucial. Uneven cross-linking can create weak spots in the film, reducing mechanical strength and barrier efficiency [80]. It is therefore necessary to develop a uniform cross-linking process for sodium alginate packaging films. 

In addition, the incorporation of functional additives—such as antimicrobial agents—into crosslinked films can alter their physical properties. This requires careful formulation to maintain an optimal balance between mechanical strength, flexibility and barrier performance [81].

#### 3.1.3. Influence of Drying Conditions, Mixing Speeds, Molecular Weight and Added Proteins

In addition to plasticizers and crosslinking, various other factors influence the mechanical properties of alginate films (summarized in Table 3).

Microscopic analyses have shown that the cross-sectional surfaces of alginate films can appear rough, with cracks forming on the surface. According to Kiattijiranon et al., these cracks are due to water loss during drying, which leads to structural stresses in the polymer network [60]. The drying conditions of the alginate films have been demonstrated to exert an influence on the films’ plasticity. However, this assertion is not universally accepted. One study suggests that single-stage drying processes at high temperatures of 80 °C should be favored because it is convenient and increases film plasticity [82]. Another study states that high drying temperatures of the films reduce the plasticizing effect because the polymer chains are shortened, leading to an increase in the elastic modulus and tensile strength. This effect is observed at temperatures up to 90 °C [66].

Another critical factor influencing the viscosity, and therefore the mechanical properties of the resultant, is the mixing speed during solution preparation. Pamies et al. reported that at high mixing speeds (5100 rpm), viscosity decreases due to the breaking of intermolecular bonds between the polymer chains [83]. Similar findings were reported by McHugh et al., who stated that shear-induced viscosity loss is generally reversible because the polymer chains can reassociate once the shear is removed [84]. However, Marcos et al. found that at very high mixing speeds (5100 rpm), the effect becomes irreversible, resulting in permanently altered solution properties. Marcos et al. also demonstrated that higher mixing speeds produce lower-viscosity sodium alginate solutions (viscosities of 0.896 Pa·s at 2000 rpm and 0.631 Pa·s at 5100 rpm), which produce stronger alginate films with improved mechanical performance [85].

In addition to the processing conditions, the molecular weight (MW) of alginate is relevant for determining viscosity and processing properties. Ureña et al. showed that alginates with higher molecular weight have higher viscosity than those with lower molecular weight. Despite their varying viscosities, no significant differences were observed in the physical properties of the two alginate types, including tensile strength, elastic modulus, and barrier properties against oxygen and water vapor. This suggests that while molecular weight influences the solution properties, it does not necessarily lead to variations in the final film properties [86].

Besides these physical parameters, the incorporation of proteins can significantly modify the properties of alginate films. When proteins are added to the alginate film, the proteins form hydrogen bonds and electrostatic interactions with Sodium alginate, which reduces the tensile strength of the films. At the same time, elongation at break, opacity, UV barrier and thermal stability increase [87,88,89].

**Table 3:** Effect of drying conditions, mixing speeds, molecular weight and added proteins on the tensile strength, young’s modulus and elongation at break of sodium alginate films.

Parameter	Effect on Tensile Strength	Effect on Young’s Modulus	Effect on Elongation at Break	Reference
drying conditions	↑ 25–90 °C drying temperature (27.20 to 46.37 MPa)	↑ 25–90 °C drying temperature (9.5 to 20.3 MPa)		[66]
molecular weight	→ 160.000–320.000 g/mol			[86]
aqueous wheat proteins	↓ 0–8% protein (11.7 ± 2.1 to 2.0 ± 0.2)		↑ 0–8% protein (17.3 ± 2.1 to 61.6 ± 12)	[87]
mixing speed			↑ 6.1–6.2% (86.9% at 2000 rpm and 92.3% at 5100 rpm)	[85]

↑: increases; ↓: decreases; →: changes to.

### 3.2. Barrier Properties

#### 3.2.1. Influence of Plasticizers and Biopolymer Blends

Glycerol is one of the most frequently utilized hydrophilic plasticizers. It has been demonstrated to enhance hydrophilicity to a considerable extent, consequently resulting in elevated WVP. In the study by Giz et al., alginate films (0 to 30 wt% glycerol/alginate) were crosslinked with different concentrations of calcium chloride solutions (0.5 to 2 wt%). The comparison of non-plasticized alginate films with films plasticized with 10% glycerol shows that the addition of glycerol has a significant effect on WVP. At 1% calcium, WVP increased by 21.89% (from 4.71 × 10^−5^ (film thickness: 0.0413 mm) to 5.00 × 10^−5^ g·m/(m^2^·day·Pa) (film thickness: 0.0385 mm)) with the addition of 10% glycerol. A similar trend was observed with 2% calcium. With the addition of 10% Gly, the WVP increased by 10% (from 3.21 × 10^−5^ (film thickness: 0.032 mm) to 3.93 × 10^−5^ g·m/(m^2^·day·Pa) (film thickness: 0.0382 mm)) [44]. Although the relative changes are measurable, their practical relevance for packaging applications is limited due to the fact that the absolute WVP values remain very high. This effect is primarily due to the presence of hydroxyl groups (-OH) in glycerol, which interact with the alginate chains, reducing the overall hydrophobicity by 16% [67]. Moreover, glycerol is highly hygroscopic, meaning that it absorbs water, further increasing water absorption and WVP [90]. Similar behavior has been observed for polyethylene glycol (PEG), which can improve the water vapor transmission rate, although it tends to induce phase separation, which may compromise the film’s homogeneity [90,91]. Jost et al. confirmed this trend and quantified the effect on gas permeability. Glycerol increased the free volume of the polymer network, significantly increasing its flexibility and oxygen permeability. For unplasticized films, oxygen permeability was approximately 0.35 cm^3^(STP)/(m^2^·d·bar). At 40 wt% glycerol (based on dry alginate), the OP exceeded 1.8 cm^3^(STP)/(m^2^·d·bar), corresponding to an increase of approximately 410%. In contrast, the OP values of sorbitol, a less efficient network-dissolving plasticizer, remained nearly constant, ranging from ~0.35 cm^3^(STP)/(m^2^·d·bar) at 30 wt% to ~0.40 cm^3^(STP)/(m^2^·d·bar) at 50 wt% [47].

In contrast, hydrophobic plasticizers, such as citrate-based plasticizers, can improve the barrier function under specific conditions. Studies have shown that at low concentrations (0.5–1% *w*/*v*), the WVTR of alginate films decreases. At higher concentrations, it is assumed that more hydrophobic ester groups are formed between the alginate chains and the citric acid, resulting in a stronger plasticizing effect, which also increases WVTR [45,48]. This demonstrates the significance of regulating the equilibrium between augmenting the barrier’s strength and modulating its plasticity, contingent on its designated application. Besides conventional plasticizers, biopolymer blends also influence the barrier properties of alginate films. As already mentioned, other polymers, such as starch, can also have plasticizing properties on alginate. Kiattijiranon et al. investigated the properties of sodium alginate/starch bilayer and multilayer films. With regard to the oxygen and water vapor barrier, the results show that the starch layers reduce the oxygen barrier (6.04 to 8.80 × 10^−14^ g·m/(m^2^·day·Pa)). Still, the oxygen barrier properties are better than those of EVOH (OP = 6.57 × 10^−14^ to 3.02 × 10^−13^ g·m/(m^2^·day·Pa)). Additionally, the water vapor barrier increases with the addition of starch (7.65 × 10^−5^ to 1.32 × 10^−4^ g·m/(m^2^·day·Pa)) due to its hydrophilic properties, reducing the effectiveness of the film as a moisture barrier [60].

Similarly, Zharkevich et al. discovered that a copolymer film consisting of 50% starch and 50% alginate has a medium barrier (5–15% undecane migration) and therefore a better barrier than PLA. Undecanes are classified as mineral oil saturated hydrocarbons (MOSH). According to EU Regulation 1935/2004, these substances are subject to strict migration limits when used in food contact applications [92]. Alginate films could therefore also be used as a coating on recycled cardboard [93].

In addition to the chemical composition of alginate films, the surface properties of alginate films are also relevant for their printability and coating performance. The main factor affecting these properties is the contact angle, which indicates the wettability of a surface [94].

One of the key parameters influencing the contact angle of alginate-based films is the concentration and type of plasticizer. Fasulo et al. investigated the surface hydrophobicity of alginate films with varying concentrations of plasticizer, comparing calcium alginate (CaAlg) films to non-crosslinked sodium alginate (NaAlg) films. In NaAlg films, an increasing concentration of glycerol–silicate (GS) plasticizer results in a continuous decrease in the contact angle (e.g., 50° at 5 wt% GS, and 39° at 35 wt% GS). This behavior is explained by the incorporation of hydroxyl-rich plasticizer molecules between the polymer chains, which disrupts intramolecular hydrogen bonding and enhances surface hydrophilicity. In contrast, CaAlg films crosslinked with 1 M calcium chloride show a non-linear behavior. At low GS concentrations (up to 10% by weight), the contact angle increases (70° at 5% by weight, 74° at 10% by weight), possibly due to increased ionic cross-linking and denser polymer packing, which temporarily reduces wettability. However, at higher GS concentrations (e.g., 50%), the contact angle decreases again (50°), likely due to saturation of the polymer matrix and the presence of excess hydroxyl groups at the surface, which increases hydrophilicity [95].

As mentioned above, according to Liling et al., deep-layer calcium crosslinking of alginate films can be prevented by too fast surface crosslinking [29]. With regard to the contact angle, the time-dependent behavior of non-crosslinked alginate films should therefore be taken into account. Mayrhofer et al. showed that uncrosslinked alginate films initially exhibit a time-dependent behavior, followed by an increase in contact angle after 5 seconds. The contact angle decreases in the first milliseconds as the water wets the surface (71.55° to 65.16°). When comparing contact angles from one to five seconds, the angle increases due to the swelling of the alginate films (66.69° to 72.24°) [94].

These aspects have a major influence on the two printing processes relevant to packaging, flexo and offset. In offset printing, the substrate generally requires a contact angle of less than 60°, whereas in flexographic printing, a range of 69° to 90° is acceptable [96,97]. The plasticizer and crosslinker concentration must therefore also be adapted to the respective printing process. At the same time, the time-dependent swelling behavior of non-crosslinked alginate inks influences adhesion in the printing process, which can reduce the quality and durability of the printed materials.

To address the issue of too high contact angles, Azucena Castro-Yobal et al. investigated the incorporation of oleic acid into alginate films, particularly in combination with glycerol, as an approach to improve their hydrophobicity. According to Azucena Castro-Yobal et al., oleic acid is an effective additive for increasing the hydrophobicity of alginate films when used in conjunction with glycerol. Oleic acid increases the hydrophobicity of a pure alginate film without additives and cross-linking by 12% compared to the pure alginate matrix. Adding both the hydrophobic oleic acid and the hydrophilic glycerol to the alginate solution leads to a synergistic behavior because the oleic acid has both a non-polar and a polar region due to the chemical structure of the carboxyl groups. The hydrophobicity is increased by 40%. Pure alginate shows a contact angle of 50°, but with oleic acid and glycerol (non-crosslinked), this drops to 30°. The combination of glycerol, oleic acid and CaCl_2_ changes the hydrophobicity further to 33°, as functional groups act both as binding sites and hydrophilic regions. An important finding from the work of Azucena Castro-Yobal et al. is that an opposite effect occurs from a glycerol concentration of 0.25%, which reduces the hydrophobic properties again. With regard to WVP, it is noticeable that the addition of glycerol and oleic acid increases the number of polar groups in the matrix, which increases the transport of water molecules through the matrix. As a result, the WVTR also increases with increasing glycerol and oleic acid content [75].

Plasma treatment has also been shown to further improve the surface properties of alginate films. As confirmed by the investigations of Sani et al., plasma increases the contact angle by modifying the surface roughness, thereby enhancing adhesion properties and wetting behavior [98].

All exact data on the influence of plasticizers on the barrier properties of alginate films is presented in the summary Table 4.

#### 3.2.2. Influence of Crosslinking

Increasing the crosslinking concentration reduces the WVP because the film forms a highly crosslinked network in certain areas, improving the barrier properties (summarized in Table 5). At the same time, a longer crosslinking time contributes to a reduction in WVP, but for a different reason. Over time, the internal structure of the film begins to dissolve due to prolonged exposure to the crosslinking solution, leading to structural degradation and increased permeability. Film thickness also plays a crucial role in crosslinking efficiency. For the same crosslinking concentration, thinner alginate films tend to have a lower WVP because the crosslinking saturates more quickly, resulting in a higher overall degree of crosslinking compared to thicker films [29].

The effectiveness of different calcium-based crosslinkers in improving the barrier properties of alginate films also varies. Studies by Jost et al. show that CaCl_2_ crosslinked films do not exhibit a significant reduction in WVP compared to the other calcium-based crosslinkers, calcium hydrogen phosphate (CaHPO_4_) and calcium carbonate (CaCO_3_). Jost et al. further demonstrate that the high WVP in CaCl_2_-crosslinked films, especially at higher Ca^2+^ concentrations, could be due to rapid crosslinking reactions that create structural inhomogeneities and prevent the formation of a uniformly dense polymer network. In contrast, CaHPO_4_ reduced WVP and oxygen permeability (OP) of the films the most, resulting in homogeneous films with improved moisture barrier properties. CaHPO_4_-crosslinked films exhibited a WVP of 37.9 ± 3.0 g·100 μm/m^2^·d·mbar and an OP of 0.08 ± 0.01 cm^3^·100 μm/m^2^·d·bar, compared to 78.7 ± 6.8 and 0.18 ± 0.01, respectively, for CaCl_2_-crosslinked films. In comparison, CaCl_2_ only reduced WVP to a limited extent, as did CaCO_3_, which also showed the lowest oxygen barrier performance. These differences can be explained by the structural uniformity of the CaHPO_4_ crosslinked films, which provides a uniform barrier throughout the film [55].

Due to their increased WVP of 78.7 ± 6.8 g-100 μm/m^2^-d-mbar (≈ 78.7 g/m^2^-d) and moderate oxygen permeability (OP) of 0.18 ± 0.01 cm^3^-100 μm/m^2^-d-bar, CaCl_2_-crosslinked films are primarily suitable for applications with low sensitivity to moisture and oxidation, such as short-term packaging applications. CaHPO_4_-crosslinked films, in contrast, with a WVP of 37.9 ± 3.0 g-100 μm/m^2^-d-mbar (≈ 37.9 g/m^2^-d) and an OP of 0.08 ± 0.01 cm^3^-100 μm/m^2^-d-bar, meet the requirements of more sensitive products such as ketchup and sauces, as well as snacks and nuts [55,99].

**Table 5:** Effect of different crosslinking agents on water vapor permeability and oxygen permeability of sodium alginate films.

Crosslinking Type	Effect on WVP	Effect on OP	Reference
Ca^2+^-crosslinked	↓ (3.96 × 10^−6^ g·m/(m^2^·day·Pa) from uncrosslinked film to 2.84 × 10^−6^ g·m/(m^2^·day·Pa) of 5% CaCl_2_ (*w*/*v*))		[29]
CaHPO_4_-crosslinked	↓ (6.62 × 10^−7^ g·m/(m^2^·day·Pa) from uncrosslinked film to 3.79 × 10^3^ g·m/(m^2^·day·Pa))	↓ (1.86 × 10^−13^ g·m/(m^2^·day·Pa) from uncrosslinked film to 1.06 × 10^−13^ g·m/(m^2^·day·Pa))	[55]

↑: increases; ↓: decreases.

### 3.3. Long-Term Stability

The paucity of long-term studies in this area is particularly salient in the context of alginate films’ use in packaging. Most research focuses on short- to medium-term evaluations of their mechanical, thermal, and barrier properties under controlled conditions [100,101,102,103]. However, understanding how alginate films behave over extended periods, particularly under varying environmental conditions, is crucial for their industrial applicability.

Thermal stability is one factor influencing the durability of alginate films. Konwar et al. showed that divalently cross-linked alginates have a higher thermostability than films that are trivalently cross-linked (Fe^3+^ and Al^3+^) [104]. The thermal properties of the polymer depend strongly on the chain mobility of the macromolecules, which is ultimately influenced by factors such as intra- or intermolecular forces, cross-linking density, etc. [105,106]. In calcium cross-linked films, decomposition starts at 227 °C. However, at 200 °C, a weight loss of 17.5% has been observed, which may be pertinent to the drying process in the context of large-scale production. For iron cross-linked films, the decomposition temperature is 195 °C or 20% [104]. In addition, the plasticizers used also influence the thermal degradation. Alginate films with glycerol and CaCl_2_ remain stable up to a temperature of approx. 60 °C. Higher temperatures (above 60 °C) lead to degradation, especially in the presence of oleic acid and high plasticizer concentrations (above 1% *w*/*v*). This must be taken into account with regard to hot filling [75].

Long-term heat exposure further affects the structural integrity of alginate films. Soazo et al. showed that heating for more than 8 minutes at 180 °C leads to a reduction in dry matter content, while the modulus of elasticity begins to decrease after 4 minutes. The longer the heat treatment, the more brittle the material becomes. SEM micrographs of the study show that the heated films had a more inhomogeneous structure than the unheated films. The heat treatment caused the films to dehydrate, making them thinner [107].

The impact of acidic substances on the long-term thermal stability of alginate films has also been studied. According to Zhou et al., the addition of acidic substances can improve the tensile strength and hydrophobicity as well as the barrier properties because the acids can interact with the sodium alginate hydrogen bonds. While these modifications improve some functional properties, they also reduce thermal stability. The onset of thermal degradation of alginate films occurs at 192 °C in the absence of acid treatment, whereas this process initiates at 174 °C in the presence of acid treatment. This finding suggests that acids can expedite the process of thermal degradation [108].

In addition to thermal and mechanical factors, humidity levels can impact the long-term performance of crosslinked alginate films. While crosslinking enhances water resistance, prolonged exposure to high humidity or temperature can lead to a gradual deterioration of barrier properties, limiting their effectiveness in certain packaging applications [17].

The interplay between temperature and humidity can lead to complex changes in the film structure, affecting properties such as water vapor permeability and mechanical strength. For example, alginate films exposed to high-humidity conditions retained more water, which can alter their dimensional stability and mechanical characteristics over time [109].

Due to their biopolymeric nature, alginate films can serve as a substrate for microbial growth, especially in humid environments. Untreated alginate films have a propensity to become contaminated with mold and bacteria, thereby reducing their viability for use in packaging applications. To counteract this, various antimicrobial additives have been studied. For instance, a study showed that alginate films enriched with watercress oil (WCO) significantly inhibited the growth of foodborne pathogens [110]. Moreover, essential oils, such as those from oregano, eucalyptus, and citronella, as well as plant extracts and nanoparticles (e.g., ZnO), have also been shown to enhance the antimicrobial properties of alginate films. These modified films exhibit effectiveness against a broad spectrum of bacteria, including both Gram-positive and Gram-negative strains. Guzmán-Pincheira et al. demonstrated that incorporating oregano essential oil into alginate-chitosan films enhanced their antimicrobial activity, making them more suitable for active food packaging [111]. Yoncheva et al. also reported that oregano essential oil enhances the antimicrobial efficacy of alginate-based systems [112]. Motelica et al. demonstrated that alginate films loaded with citronella essential oil can inhibit the growth of foodborne pathogens, suggesting their potential use in active packaging [113]. Kowalonek et al. demonstrated that incorporating zinc oxide nanoparticles into alginate films significantly enhances their antimicrobial activity [114].

Overall, the long-term stability of alginate films is determined by a balance between crosslink density, chain mobility, and residual moisture content. Increased crosslink density has been shown to enhance tensile strength while concomitantly reducing water absorption. However, this increased density can also impose limitations on elongation at break and can expedite the formation of microcracks during the processes of drying or when there is a change in humidity. In contrast, films with higher plasticizer content retain their flexibility but exhibit higher WVP and OP due to increased free volume. Long-term mechanical and barrier performance therefore requires a balanced medium network density that maintains cohesion while tolerating limited molecular movement. This ensures the durability of alginate films under real storage conditions.

**Figure 1:**
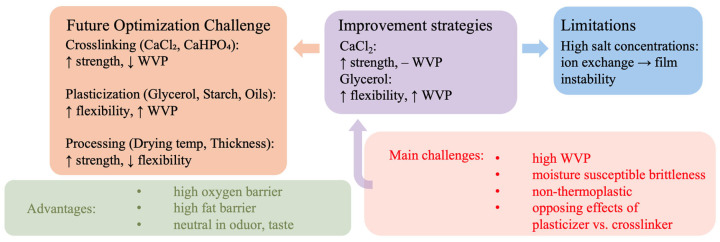
Schematic summary of the main challenges and optimization strategies for alginate-based cast films.

As discussed in Section 3.1, Section 3.2 and Section 3.3, the mechanical, barrier, and long-term stability of alginate films are governed by complex interactions between formulation and processing. The summary in Figure 1 provides a visual representation of these interrelations and delineates the current challenges and optimization directions for future research.

## 4. Possible Applications of Alginate Films in the Food Industry

Alginate films have been widely investigated for food packaging, but their susceptibility to electrolyte-induced swelling, low antimicrobial activity, weak water-vapor barrier, and poor UV protection still pose significant challenges [100,115,116,117,118,119,120].

Alginate films show no pH-dependent swelling behavior. While changes in pH resulting from microbial activity can influence the rate of food spoilage, the presence of electrolytes exerts a considerably more pronounced effect on the swelling behavior of alginate films. With a sodium chloride concentration of 0 to 2 M, the water solution in contact with alginate films favors ion exchange with the alginate film. Calcium ions are released from the egg carton structure, loosening the network and allowing more water to be adsorbed. This causes the film to dissolve. Accordingly, the use of alginate films as packaging materials should be avoided for foods with high salt content. The 2 M NaCl solution used in the experiments corresponds to approximately 11.7 g of NaCl per 100 g of solution, representing very strong laboratory conditions [100]. In practice, films could be influenced by considerably lower salt concentrations, although more slowly or only slightly, especially in liquid or pasty foods where the film is in continuous contact with the salt-containing phase.

A further constraint pertains to the diminished antimicrobial potency exhibited by alginate films. In contrast to other biopolymers that possess intrinsic antibacterial properties, alginate has been demonstrated to be ineffective in impeding the proliferation of microbes on food surfaces. Therefore, the addition of functional additives such as essential oils, nanoadditives or probiotics is required to increase antimicrobial efficacy [115,116,117].

The hydrophilic properties of alginate have been demonstrated to influence the barrier functions of the material. Specifically, alginate exhibits a low water vapor barrier property. This means that moisture can easily permeate through the film. This property poses a challenge for typical moisture-sensitive products such as crisps, milk powder or coffee, where even small amounts of penetrating moisture can lead to a loss of quality. However, for products that tend to dry out, such as fresh fruit or vegetables, high WVP can also offer advantages, as it reduces condensation and thus improves microbiological stability [99,115,116,117]. Furthermore, alginate films offer little protection against UV radiation, which can lead to color changes, nutrient degradation, and the formation of free radicals, which in turn can affect the quality of packaged foods [117,118,119].

Another important factor limiting the widespread commercial use of alginate films in food packaging is their inconsistent performance under varying environmental conditions. Fluctuations in temperature and humidity during storage have been demonstrated to induce instability in the properties of the film, thereby compromising its mechanical strength and barrier function [121].

Despite these challenges, numerous studies have investigated various applications of alginate films in food preservation. One of the most researched areas is their use in extending the shelf life of fresh produce. Numerous studies have demonstrated that the application of alginate-based coatings or the use of alginate films can slow the ripening process and minimize moisture loss in fruits and vegetables. This results in a prolonged shelf life and a reduction in spoilage [122,123,124,125,126]. Untreated alginate films can serve as a substrate for microbial growth under humid conditions. However, studies have shown that the application of alginate-based coatings on fresh products such as strawberries can significantly delay mold development. For example, strawberries coated with sodium alginate and calcium chloride remained mold-free for up to 15 days at 4 °C, while uncoated samples showed visible mold after just one week [127].

For meat, fish, and poultry, alginate films could serve as protective packaging by preventing oxidation, moisture loss, and microbial contamination. Nonetheless, owing to their inadequate antimicrobial activity and insufficient water vapor barrier properties, meat, fish, and poultry applications necessitate functional modification. The addition of essential oils, nanoadditives, or lactic acid bacteria has been investigated as a method to improve the preservation properties of alginate-based packaging for perishable protein-containing foods. Without such modifications, alginate films alone do not provide sufficient protection against microbial spoilage [8,120,128,129,130,131].

## 5. Conclusions

The results of this study demonstrate that alginate films have the potential to achieve a level of performance that is comparable to that of conventional food packaging films. From a mechanical perspective, calcium alginate films with a thickness of 12.6 µm exhibit tensile strengths of approximately 130 MPa, comparable to materials such as PET [29,132]. However, the mechanical properties of these materials are found to be highly dependent on the thickness of the film, which must be taken into account when assessing their performance.

However, the mechanical properties are contingent upon the film thickness and the uniformity of the cross-linking, which must be considered in the evaluation.

The requirements for applications such as food packaging are notably stringent with regard to barrier properties. While alginate films have been shown to achieve superior oxygen permeability values, often exceeding those of EVOH (typically ranging from 1000 to 1500 g/(m^2^·d), at 24 ± 1 °C, 38 ± 1% RH) their high water vapor permeability remains a significant constraint [44,60,99]. Although the high water uptake is disadvantageous for moisture-sensitive products, it can prevent internal condensation or drying out and extend the freshness of products such as fruit and vegetables.

Cross-linking and plasticization have been identified as key factors in the targeted modification of these properties. Calcium chloride has been demonstrated to enhance tensile strength; however, concentrations exceeding 2% (*w*/*v*) result in the formation of uneven networks and surface cracks, which compromise the performance of the barrier [24,25,26,29].

Plasticizers, such as glycerin, enhance flexibility; however, due to their hygroscopic nature, they compromise barrier efficacy and long-term stability [45,47,64,65,66]. The incorporation of biopolymer-based plasticizers, such as starch, also enhances elasticity but results in a substantial augmentation of water vapor permeability [15,60]. In contrast, hydrophobic additives, such as tributyl citrate or oregano oil, have been shown to enhance the moisture barrier properties of the material. However, these additives frequently result in a reduction in mechanical strength [68]. A balanced overall performance of an alginate film therefore requires careful coordination of the plasticizer system with the respective degree of cross-linking.

There are still limitations in terms of practical applicability, particularly for packaging foods with high salt content, where ion exchange with sodium can destabilize the cross-linking structure and lead to partial dissolution [100].

In summary, the central challenge is not to improve individual material properties, but to achieve a stable balance between mechanical strength, barrier performance, and processability. The biggest research gap is in converting laboratory-optimized formulations into process-stable films that retain their properties under industrial conditions. Particular attention should be paid to moisture management, drying control, and the interaction of plasticizers and crosslinking systems, as these parameters are critical in determining long-term performance [95]. From an application-oriented perspective, multilayer or hybrid structures represent the most promising approach. In these systems, alginate serves as an effective oxygen and fat barrier.

In summary, the primary strengths of alginate can be attributed to its excellent oxygen barrier properties, renewability, and biocompatibility. Conversely, its primary weaknesses are moisture sensitivity and limited flexibility. A holistic development approach that combines formulation, processing, and multilayer strategies offers the most promising path to economically viable, plastic-free packaging solutions.

## Figures and Tables

**Table 2:** Effect of different crosslinking agents on the tensile strength and elongation at break of sodium alginate films.

Crosslinking Type	Effect on Tensile Strength	Effect on Elongation at Break	Reference
Ca^2+^-crosslinked	↑ (40,209 MPa not crosslinked to 134,580 MPa) ↓ over 2% concentration	↑ (3.64% not crosslinked to 5.55%) ↓ after 2 min crosslinking time	[29]
citric acid-crosslinked	↓ from 22 MPa (control) to 9.3 MPa (1% CA) and to 7.5 MPa (2% Ca)	↑ from 4% (control) to 15% (1% Ca) and to 23% (2% Ca)	[48]
Al^3+^-crosslinked	↑ (40,209 MPa not crosslinked to 81,421 MPa)	↓ (3.64% not crosslinked to 2.92%)	[29]

↑: increases; ↓: decreases.

**Table 4:** Effect of different plasticizers on water vapor permeability, oxygen permeability, and contact angle of sodium alginate films.

Plasticizer/Additives	Effect on WVP	Effect on OP	Effect on Contact Angle	Reference
Glycerol	↑ from 4.65 × 10^−10^ g·m/(m^2^·day·Pa) at 0% glycerol to 4.94 × 10^−10^ g·m/(m^2^·day·Pa) at 10% glycerol (difference not reported as significant)		↓ e.g., 50° at 5% Glycerol, 39° at 35% Glycerol	[44,95]
Glycerol (40 wt% *w*/*w* dry alginate)		↑ 410% (from ~0.35 to ~1.8 cm^3^(STP)·100 µm/(m^2^·d·bar))		[47]
PEG	↓ 14.69% (1.82 × 10^−2^ g·m/(m^2^·day·Pa) by Glycerol, compared to 1.56 × 10^−2^ g·m/(m^2^·day·Pa))			[90]
Citric acid	↓ up to 34% (from 7.97 × 10^4^ g·m/(m^2^·day·Pa) to 5.24 × 10^4^ g·m/(m^2^·day·Pa) with 1% Citric acid)			[45,48]
Starch	↑ 73.03% (from 7.65 × 10^−5^ g·m/(m^2^·day·Pa) to 1.32 × 10^−4^ g·m/(m^2^·day·Pa))	↓ 45.65% (from 8.80 × 10^−14^ to 6.04 × 10^−14^ g·m/(m^2^·day·Pa))		[60]
Oleic acid	↑ (from 4.5 × 10^2^ g·m/(m^2^·day·Pa) without oleic acid to 6.00 × 10^2^ g·m/(m^2^·day·Pa) with 10% oleic acid)		↓ 40% (pure alginate 50°, with oleic acid 30°)	[75]
Sorbitol (50 wt% *w*/*w* dry alginate)		↑ ≈ 14% (from ~0.35 to ~0.40 cm^3^(STP)·100 µm/(m^2^·d·bar))		[47]

↑: increases; ↓: decreases.

## Data Availability

No new data were created or analyzed in this study.

## Abbreviations

The following abbreviations are used in this manuscript:

WVP	Water Vapor Permeability
OP	Oxygen Permeability
WVTR	Water Vapor Transmission Rate
TS	Tensile Strength
Ca^2+^	Calcium Ion
CaCl_2_	Calcium Chloride
CaHPO_4_	Calcium Hydrogen Phosphate
CaCO_3_	Calcium Carbonate
PEG	Polyethylene Glycol
OEO	Oregano Essential Oil
EVOH	Ethylene Vinyl Alcohol
MW	Molecular Weight
DSC	Differential Scanning Calorimetry
SEM	Scanning Electron Microscopy
PET	Polyethylene Terephthalate
NaAlg	Sodium Alginate
CaAlg	Calcium Alginate
SA	Sodium Alginate
PPWR	Packaging and Packaging Waste Regulation (EU)
SUPD	Single-Use Plastics Directive (EU)
MOSH	Mineral Oil Saturated Hydrocarbons
UV	Ultraviolet
LAB	Lactic Acid Bacteria
WCO	Watercress Oil
ZnO	Zinc Oxide
RH	Relative Humidity
MPa	Megapascal
